# Retraction: The impact of different plant extracts on population suppression of *Helicoverpa armigera* (Hub.) and tomato (*Lycopersicon esculentum* Mill) yield under field conditions

**DOI:** 10.1371/journal.pone.0272432

**Published:** 2022-08-17

**Authors:** The PLOS ONE Editors

The *PLOS ONE* Editors retract this article [1] because it was identified as one of a series of submissions for which we have concerns about authorship, competing interests, and peer review. We regret that the issues were not addressed prior to the article’s publication.

SA, YL, MM, MS, and ATKZ did not agree with the retraction. IUH, WA, MZS, MMK, YN, TF, OM, and MJA either did not respond directly or could not be reached.
